# The Retinal Vessel Density as a New Vascular Biomarker in Multisystem Involvement in Fabry Disease: An Optical Coherence Tomography Angiography Study

**DOI:** 10.3390/jcm9124087

**Published:** 2020-12-18

**Authors:** Gilda Cennamo, Daniela Montorio, Ciro Santoro, Sirio Cocozza, Letizia Spinelli, Teodolinda Di Risi, Eleonora Riccio, Camilla Russo, Giuseppe Pontillo, Roberta Esposito, Massimo Imbriaco, Antonio Pisani

**Affiliations:** 1 Eye Clinic, Public Health Department, University of Naples “Federico II”, 80131 Naples, Italy; 2 Department of Neurosciences, Reproductive Sciences and Dentistry, University of Naples “Federico II”, 80131 Naples, Italy; da.montorio@gmail.com; 3 Department of Advanced Biomedical Sciences, Federico II University Hospital, 80131 Naples, Italy; cirohsantoro@gmail.com (C.S.); siriococozza@hotmail.it (S.C.); letspine@gmail.com (L.S.); camilla_russo@hotmail.it (C.R.); giuseppe.pon@gmail.com (G.P.); robyeire@tin.it (R.E.); mimbriaco@hotmail.com (M.I.); 4 CEINGE—Advanced Biotechnologies, 80145 Naples, Italy; lindadirisi@gmail.com; 5 Department of Public Medicine, University Federico II, 80131 Naples, Italy; elyriccio@libero.it (E.R.); antonio.pisani13@gmail.com (A.P.); 6 Department of Electrical Engineering and Information Technology, University of Naples “Federico II”, 80125 Naples, Italy

**Keywords:** Fabry disease, optical coherence tomography angiography, retinal vessel density, echocardiography, peak arterial pulmonary pressure, left atrial volume index, TSVDS

## Abstract

In this study, we evaluated the possible relationship between the changes in retinal vessel density (VD) by optical coherence tomography angiography (OCTA) and the vascular alterations involving renal, cardiovascular and central nervous systems in patients affected by Fabry disease (FD). In 50 FD patients, the retinal superficial capillary plexus (SCP) and deep capillary plexus (DCP) in macular region were evaluated by OCTA examination. The patients also underwent a brain magnetic resonance imaging scan, renal and echocardiographic examinations with quantification of systolic pulmonary arterial pressure (PAPs) and left atrial volume index (LAVi). The VD of SCP and DCP was inversely related with E/e’ ratio, LAVi, interventricular septal thickness, global longitudinal strain (GLS) and PAPs (*p* < 0.05). No relationship was found, with a multivariate analysis, between OCTA parameters and kidney function and neuroradiological signs of central nervous system involvement. OCTA could be a new vascular biomarker in FD, revealing a strong correlation between retinal capillary damage and myocardial impairment, possibly preceding both renal dysfunction and cerebrovascular involvement.

## 1. Introduction

Fabry disease (FD) is a rare X-linked lysosomal storage disorder caused by a deficit in the enzyme α-galactosidase A, with the progressive accumulation of globotriaosylceramide (Gb3) in different cells, such as vascular endothelium, smooth muscle cells, renal cells, cardiomyocytes, and neuronal cells [1,2,3,4,5].

The main causes of vascular abnormalities in FD have been considered the endothelial damage due to Gb3 deposition and the hypertrophy of the medium-caliber arteries due to increased intima-media thickness and vascular smooth muscle cell proliferation [6].

Independently from its pathophysiology, this vascular involvement leads to a progressive severe multiorgan dysfunction, as represented by cardiac involvement (with arrhythmias and left ventricular (LV) hypertrophy), central nervous system (CNS) involvement (mainly represented by the occurrence of cerebrovascular events) and renal dysfunction (causing proteinuria and impairment in glomerular filtration rate—GFR); all factors ultimately leading to premature death [2,7,8].

Being a systemic disorder, FD also affects the ocular system. Its involvement is mainly represented by the appearance of cornea verticillata, lens opacity and vascular abnormalities. With particular reference to the latter, the pathological deposition of Gb3 in both the retinal vascular endothelial cells and in the tunica media of small vessels is known to induce a decreased resistance to hydrostatic pressure, as well as an impaired vascular remodeling, leading to increased vascular tortuosity, aneurysmal dilatation and occlusive events [9,10,11,12].

The introduction of a non-invasive imaging techniques, such as optical coherence tomography angiography (OCTA), has allowed for a detailed and quantitative analysis of the retinal vascular networks, identifying microvascular changes in different retinal disorders [13,14].

Although previous OCTA studies have demonstrated significant alterations of the vessel density of superficial capillary plexus (SCP) and deep capillary plexus (DCP) in FD patients, there is currently no information about the possible association with other biomarkers of different organ involvement [15,16,17].

The aim of this observational study was to correlate OCTA parameters with other imaging biomarkers, to evaluate a possible relationship between the retinal perfusion changes and the vascular alterations involving different systems (namely, renal, cardiovascular and CNS) in FD patients at diagnosis.

## 2. Experimental Section

### 2.1. Subjects

Data from 59 FD patients were retrospectively analyzed in subjects evaluated from July 2017 to May 2018. Inclusion criteria were: genetically confirmed diagnosis of FD, 18 years of age, naïve for FD treatment. Exclusion criteria were: evidence of ocular and systemic diseases unrelated to Fabry disease, current or previous macular and retinal vascular diseases, diagnosis of glaucoma, congenital eye disease, high myopia (>6 dioptres), significant lens opacification and reduced-quality OCTA images. FD patients with advanced organ involvement were excluded from the study for possible confounding interaction. In particular, six patients were excluded because of significant renal impairment (GFR < 50 mL/min/1.73 m^2^ and/or proteinuria >1 gr/die), and three subjects were excluded because of overt diastolic heart failure, leading to a final number of 50 FD patients were included in this study (36 females, mean age 41.5 ± 15.3 years).

For all patients, signs of cardiac, renal, and CNS involvement were recorded. In particular, cardiac involvement was evaluated, according to previous reports [18], by clinical examination and a complete echocardiographic exam. Renal involvement was defined if an estimated glomerular filtration rate (eGFR) <90 mL/min and or a proteinuria > 150 mg/24 h were present. Finally, CNS involvement was evaluated as follows: for each patient for whom an MRI scan was available (41/50), images were examined in consensus by two neuroradiologists (GP and CR) to assess the presence of cerebral macrovascular events (namely stroke), while a global scale to assess the severity of vascular disease was determined according to the total MRI brain small vessel disease score (TSVDS) [19]. Patients with MRI signs of stroke or a total TSVDS ≥ 1 were considered as affected.

All subjects underwent a complete ophthalmological evaluation including the evaluation of best corrected visual acuity (BCVA) according to the Early Treatment of Diabetic Retinopathy Study (ETDRS), slit-lamp biomicroscopy, intraocular pressure measurement, fundus examination, and OCTA.

The study was approved by the Institutional Review Board of the University of Naples “Federico II” (protocol number: 109/05) and all investigations adhered to the tenets of the Declaration of Helsinki. Signed informed consent was obtained from each patient.

### 2.2. Optical Coherence Tomography Angiography

OCTA was performed by the Optovue Angiovue System (software ReVue XR version 2017.1.0.151, Optovue Inc., Fremont, CA, USA) that is based on a split-spectrum amplitude de-correlation algorithm (SSADA) and which uses blood flow as intrinsic contrast [20].

The evaluation of the macular capillary network was performed in a 6 × 6 mm scan centered on the fovea. The OCTA software automatically analyzed the macular region divided into whole image, fovea and parafovea in each vascular network of the retina: SCP and DCP, according to the ETDRS classification of diabetic retinopathy.

The AngioAnalytics™ software automatically calculated the vessel density that represents the percentage area occupied by the vessels in the analyzed region [21].

Images with a signal strength index less than 40 or residual motion artefacts, incorrect segmentation or low centration and focus were excluded.

### 2.3. Echocardiography

Within 2 weeks of the ophthalmological evaluation, patients underwent two-dimensional transthoracic echocardiography including determination of LV global longitudinal strain (GLS) with the use of commercially available equipment (Vivid E95 ultrasound scanner—Horten, Norway) equipped with a 2.5 MHz phased-array transducer and provided with a software-based beamforming algorithm. Blood pressure and heart rate were measured at the end of the echo exam. Cardiac chambers quantitative analysis was performed in agreement with 2015 recommendations [22]. LV mass was calculated by Devereux formula and normalized by height in meters to the power of 2.7. LV myocardial walls were considered to be hypertrophic when maximal wall thickness was ≥10 mm in women and ≥11 mm in men with the AFD pathogenic mutation. Left atrial volume (Simpson method in apical four-chamber and two-chamber views) was indexed to body surface area (left atrial volume index). LV diastolic function parameters were determined according to current recommendations [23]. Speckle-tracking echocardiography (STE) acquisition and post-processing (Echopac 2.02, release 34.0) were performed in apical long-axis, 4-chamber, and 2-chamber views according as previously described [18,24]. GLS was calculated by averaging all values of regional peak systolic longitudinal strain obtained in each apical.

Systolic pulmonary artery pressure (PAPs) was computed by measuring the tricuspid regurgitation (TR) peak velocity, summing an estimate of right atrial pressure (RAP) referred to the size and respiratory reactivity of the inferior vena cava (IVC): (a) normal RAP (≈5 mmHg); normal IVC size (IVC diameter < 2.1 cm) with normal inspiratory collapse (>50% decrease in IVC diameter); (b) RAP ≈ 10 mmHg: dilated IVC (diameter > 2.1 cm) or <50% collapse; (c) RAP ≈ 15 mmHg: both dilated IVC and <50% collapse; (d) RAP ≈ 20 mmHg: dilated IVC without visible collapse, as recommended [25].

### 2.4. Statistical Analysis

All statistical data were processed using standard statistical software SPSS (ver. 24.0, IBM, 21 Chicago, IL, USA). Continuous variables were confirmed for normal distribution by the Kolmogorov–Smirnov test and expressed as mean values ± standard deviation (SD). Differences between the two groups in continuous variables were analyzed using independent t-test for normal distribution and Mann–Whitney U test for non-normal distribution.

From the enrolled subject, one eye was randomly selected for the subsequent analysis, leaving a total number of 50 eyes. Pearson’s correlation was used to evaluate univariate correlates of a given variable. Multivariable linear regression analyses were performed to examine the independent correlates between superficial capillary plexus (SCP) and of the deep capillary plexus (DCP) values and biomarkers of other systems involvement. The null hypothesis was rejected at *p* < 0.05.

## 3. Results

Demographic data and clinical parameters of the FD are shown in Table 1.

Most patients had normal renal function and proteinuria levels, as shown in Table 1.

All patients were free from cardiac symptoms/signs. LV standard echocardiographic and STE assessment are reported in Table 2.

### Univariate and Multivariate Associations

**Figure 1:**
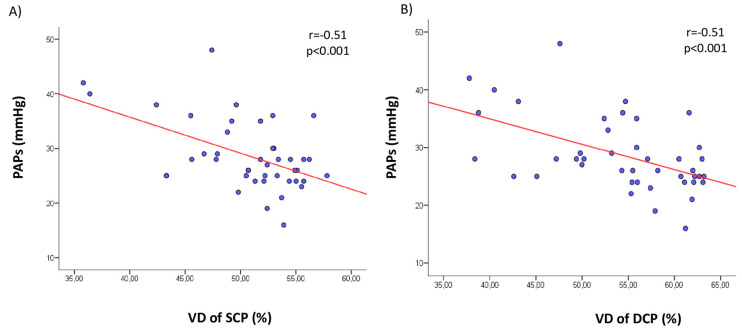
Scatter plots showing significant correlations between the vessel density (VD) of retinal superficial capillary plexus (SCP) in whole image and systolic pulmonary artery pressure (PAPs) (**A**) and between the vessel density (VD) of retinal deep capillary plexus (DCP) in whole image and PAPs (**B**).

In the pooled FD population, the vessel density of SCP and DCP in whole image was inversely related with E/e’ ratio, LAVi, interventricular septal thickness, GLS and PAPs (Table 3 and Figure 1). These correlations remained significant after considering foveal and parafoveal regions of superficial and deep plexus separately (*p* < 0.05).

Patients with neuroradiological signs of CNS involvement (n = 6/11.5%) showed lower vessel density of SCP compared to those without (46.2 ± 8.12 vs. 51.7 ± 3.73; *p* < 0.001).

No relationships were found between both SCP and DCP parameters and markers of kidney function such as GFR (r = 0.06; *p* = 0.66 and r = 0.13; *p* = 0.34, respectively) and proteinuria (r = 0.04; *p* = 0.75 and r = −0.05; *p* = 0.71, respectively).

By a multivariable regression performed in the pooled FD population, after adjusting for age, interventricular septal thickness and a parameter of early systolic dysfunction such ad GLS and PAPs were independently associated with vessel density of SCP in whole image (standardized β coefficient = −0.553, *p* < 0.001) and DCP in whole image (standardized β coefficient = −0.474, *p* = 0.004) (Table 4).

## 4. Discussion

In this study, we found that the retinal microvascular alterations, as evaluated by a non-invasive technique such as the OCTA, correlate with microvasculature damages in other organs, suggesting a possible role of this measure as an early biomarker of tissue damage in FD.

Indeed, when assessing correlations between OCTA values and other systemic findings, we found a significant negative correlation between the reduced retinal vascular networks and the increased PAPs, LAVi and E/e’ ratio. These echocardiographic parameters are known to represent sensible biomarkers of LV diastolic dysfunction and LV diastolic filling pressure, an early index of myocardial damage [26,27]. Indeed, in FD patients, the glycosfingolipids accumulate not only in endothelium, with consequent vascular dysfunction, but also in cardiomyocytes [28]. This accumulation promotes inflammation, with increased extracellular matrix deposition, hypertrophy and fibrosis, ultimately resulting in LV function impairment and left atrial dilation [6,29]. It is important to remember that LV diastolic dysfunction plays a crucial role in the development of cardiac symptoms in FD patients, leading, in some cases, to the development of heart failure with preserved LV ejection fraction. Indeed, diastolic dysfunction appeared to correlate with late gadolinium enhancement and consequently to endomyocardial fibrosis in patients with FD [30] On the other hand, impairment of coronary microvascular function is also an important feature of FD cardiomyopathy. Indeed, coronary microvascular dysfunction has been demonstrated in FD patients with LV hypertrophy [31], with hallmarks related to the increase in LV mass (e.g., reduced capillary density or extravascular compression forces) that could be considered as mechanisms underlying coronary blood flow impairment. However, mechanisms related to FD, such as endothelial dysfunction due to Gb3 storage, nitric oxide pathway dysregulation, or microvascular remodeling could directly affect microvascular function. Tomberli et al. [32] found that coronary microvascular function was markedly impaired in FD patients, irrespective of LV hypertrophy and gender, and concluded that coronary blood flow impairment may be an early sign of cardiac involvement as it precedes the development of cardiac hypertrophy. It is worth to underline that coronary microvascular dysfunction and chronic hypoperfusion, due to Gb3 accumulation in endothelial cells, may also play a crucial role in inducing impairment of myocardial function. Thus, the association between retinal vessel density and LV diastolic dysfunction or subclinical systolic function impairment is not surprising. Among LV diastolic function parameters, PAPs showed the best correlation with either vessel density of SCP or DCP. Along with diastolic dysfunction, a role of pulmonary microvascular dysfunction in contributing to the increase in PAPs cannot be ruled out.

On the other hand, the lack of correlation of retinal vessel density of SCP and DCP with parameters of kidney function is, although disappointing, not surprising. Indeed, renal vessel disease is a recognized key feature of FD-related nephropathy. However, renal involvement is characterized by the accumulation of Gb3 in virtually all cell types, including endothelial and epithelial glomerular cells, podocytes and tubular cells. Interstitial fibrosis with interstitial infiltrate of inflammatory cells is known to play a role in kidney damage development. Tøndel et al. [33], by performing kidney biopsies in nine children, found out that interstitial fibrosis was already present in these young patients. Therefore, FD patients may present glomerular and tubular disorders late in the disease development, being absent in the early stages where vascular disease is predominant. In this study, the absence of significant correlation between OCTA and renal function is likely due to the normal renal function and to the absence of proteinuria observed in FD patients.

Similarly, the overall absence of significant correlation between the different neuroradiological parameters here investigated and OCTA values can be also interpreted as a further indication of the role of the latter as an early biomarker of the disease. Indeed, the presence of neuroradiological signs suggestive of cerebrovascular involvement, either WMH, stroke or microbleeds, are known to be irreversible manifestations of macrovascular CNS damage, occurring at a late phase of the disease [34]. On the other hand, metrics of microstructural damage, such as those obtained via advanced MRI techniques such as the analysis of diffusion tensor imaging (DTI), are known to provide early information about the microstructural status of brain structures, somehow preceding the presence of macrostructural damage [35,36]. For this reason, future studies correlating indices of microstructural damage and OCTA values are suggested, to further expand and confirm the possible role of these metrics as possible biomarker of the disease.

These alterations of the retinal vessel density of SCP and DCP at OCTA have been hypothesized to reflect vessel walls remodeling due to Gb3 accumulation in smooth muscle and endothelial cells [4]. In line with this speculation, a previous study showed a significant decreased vessel density of the SCP—a sign of a reduced blood flow due to increased intima-media thickness affecting the distensibility of the vessels and the lumen diameter. The vessel density of the DCP was significantly increased, possibly due to a vascular compensation mechanism to support the reduced SCP [15]. Similar results were found in a different study that showed a reduced vessel density in both retinal vascular networks [16], while another work showed the presence of vascular tortuosity in the superficial plexus, with vascular rarefaction areas in the deep plexus coupled to increased enlargement of foveal avascular zone [17]. Furthermore, it has also been hypothesized a possible increased inflammatory and platelet activity that could trigger thrombotic events in FD patients, which are known to be frequent in this condition [37,38].

The retinal and cerebral small vessels share similar embryologic origins, anatomical features, and physiological properties; therefore, the retinal vessels may serve as a research model to detect subclinical cerebrovascular changes associated with FD [39,40,41,42,43,44,45,46].

The main limitations of this study are represented by the small sample size and the absence of patients with advanced FD stages.

Although characterized by these limitations, our study shows the presence of a correlation between the retinal perfusion values and echocardiographic parameters of diastolic dysfunction in FD patients, while other vascular parameters known to be late findings in this condition showed no correlation with OCTA values. This parallel impairment of both vessel density in the macular region and myocardial function supports the hypothesis that OCTA could represent a valid biomarker to predict the early cardiovascular damages in FD. This technique could identify patients with subclinical changes, allowing early interventions and prevent the development of more severe clinical consequences.

## Figures and Tables

**Table 1:** Clinical features of the study population.

Variable	FD (*n* = 50) Mean ± SD (Range)
Gender (F/M)	36/14
Age (years)	41.5 ± 15.3 (14–79)
BMI (kg/m^2^)	25.8 ± 4.8 (16.8–38.9)
Systolic BP (mmHg)	120.4 ± 17.5 (90–160)
Diastolic BP (mmHg)	74.5 ± 10.3 (60–100)
Heart rate (bpm) Proteinuria (mg/24 h) GFR (mL/min/1.73 m^2^)	67.5 ± 9.4 (47–92) 162.6 ± 144.7 (0–600) 101.3 ± 17.9 (55.0 ± 140)

BMI = body mass index, BP = Blood pressure, GFR: glomerular filtration rate.

**Table 2:** Standard and speckle tracking echocardiography data of the study population.

Variable	aADF (*n* = 50) Mean ± SD (Range)
IVS thickness (cm)	1.00 ± 0.33 (0.6–2.3)
LV mass index (g/h ^2.7^)	43.9 ± 21.5 (23.1–124.9)
RWT	0.42 ± 0.18 (0.22–1.24)
E/a ratio	1.26 ± 0.50 (0.19–2.6)
E/e’ ratio	8.3 ± 3.7 (1.21–16.8)
PAPs (mmHg)	28.7 ± 6.4 (16–48)
LAVi (mL/m^2^)	32.1 ± 9.7 (15–60)
LV EF (%)	61.7 ± 5.5 (44–73)
LV GLS (%)	19.4 ± 3.73 (11–28.2)

IVS: interventricular septum; LV: left ventricle; RWT: relative wall thickness; E/a ratio: early mitral inflow peak velocity to late mitral in low peak velocity ratio; E/e’ ratio early mitral inflow peak velocity to early diastolic mitral annulus peak velocity ratio; PAPs = systolic pulmonary artery pressure; LAVi: left atrial volume index; EF: ejection fraction; GLS: global longitudinal strain.

**Table 3:** Univariate correlation between superficial capillary plexus (SCP) and deep capillary plexus (DCP) in whole image with standard and advanced echocardiographic parameters.

Dependent Variable	Covariate	Β Coefficient	*p*
SCP	E/e’ ratio	−0.32	<0.03
	LAVi (mL/m^2^)	−0.48	<0.001
	PAPs (mmHg)	−0.51	<0.0001
	IVS thickness (cm)	−0.32	<0.04
	GLS (%)	0.31	<0.04
DCP	E/e’ ratio	−0.29	<0.05
	LAVi (mL/m^2^)	−0.45	<0.002
	PAPs (mmHg)	−0.51	<0.0001
	IVS thickness (cm)	−0.34	<0.03
	GLS (%)	0.34	<0.02

**Table 4:** Independent determinants of vessel density of SCP and DCP in whole image by multiple linear regression analyses.

Dependent Variable	Covariate	Β Coefficient	*p*
SCP	Age (years)	−0.027	0.858
	IVS thickness (cm)	−0.301	0.213
	GLS (%)	−0.151	0.535
	PAPs (mmHg)	−0.553	<0.001
DCP	Age (years)	−0.059	0.709
	IVS thickness (cm)	−0.164	0.514
	GLS (%)	0.018	0.945
	PAPs (mmHg)	−0.474	0.004

Cumulative R2 = 0.383, SEE = 4.238 %, *p* = 0.003. Cumulative R2 = 0.321, SEE = 6.829 %, *p* < 0.01.
